# Peak Age of Information Analysis in Systems with Multiple Time-Correlated Traffic Streams

**DOI:** 10.3390/s25051440

**Published:** 2025-02-26

**Authors:** Varvara Manaeva, Elena Zhbankova, Ekaterina Markova, Konstantin Samouylov

**Affiliations:** 1 Department of Probability Theory and Cybersecurity, People’s Friendship University of Russia Named After Patrice Lumumba, 115419 Moscow, Russia; zhbankova_ea@rudn.ru (E.Z.); markova_ev@rudn.ru (E.M.); samuylov_ke@rudn.university (K.S.); 2 Federal Research Center “Computer Science and Control” of the Russian Academy of Sciences, 119333 Moscow, Russia

**Keywords:** AoI, Age of Information, 5G, Internet of Things

## Abstract

Nowadays, Internet of Things (IoT) is one of the most dynamically evolving services in the 5G ecosystem. In industrial IoT (IIoT), this service can be utilized to deliver state updates of various equipment to the remote control center for further coordination and maintenance. As a result, one of the critical metrics of interest for such a service is the Age of Information (AoI) and its upper bound—peak AoI (AoI)—characterizing the freshness of information about the state of the systems. In spite of significant attention, these metrics received over the last decade, only little is known regarding the PAoI performance of a single source (e.g., sensor) in the presence of competing traffic from other sources in queuing systems. On top of this, models with batch arrivals and batch services that can be effectively used to represent service performance in modern cellular systems such as 5G New Radio are lacking. In our study, we consider a cellular air interface representing it as a queuing system (QS) in discrete-time with batch arrivals and service and investigate performance of a single (tagged) source in presence of competing traffic from other sources having the same priority, where all the sources are modeled using the switched Poisson process (SPP) characterized by sophisticated correlational properties. We also investigated the impact of several service disciplines on the performance of the tagged source including first-come–first-served (FCFS), last-come–first-served (LCFS), random, and priority-based service. Our results illustrate that, although the qualitative behavior of the mean PAoI is different for different service disciplines, the optimal value of PAoI is insensitive to the choice of the service order. On top of this, we observed that introducing a priority in service to one of the flows may drastically affect the performance of other flows even when the overall load contribution of a single flow is rather limited. Our observations can be utilized to design packet scheduling strategies for 4G/5G cellular systems carrying traffic of state update applications.

## 1. Introduction

The Internet of Things (IoT) is a type of communication system that includes so-called “things” and the communication networks connecting them with each other and processing units [1]. The term “things” in IoT describes any physical object that can be augmented with sensors and communication units to provide remote monitoring, status updates, manipulation, and control [2].

IoT has recently found a plethora of applications in various fields, especially in the industrial sector. As a matter of fact, industrial Internet of Things (IIoT) is nowadays classified in its own category of IoT applications [3,4]. The IIoT can be utilized in energy grids, network load control, smart factories, crop monitoring, and various other industries. The end user equipment (UE) in IIoT acquires and analyzes data from connected units, different locations, sensors, and various other types of “things” in order to maintain, control, or manipulate the workflow remotely and automatically.

To adequately process the information in a remote control center (CC) and make timely decisions, the information should be as relevant (up-to-date) as possible. In this case, the Age of Information (AoI) metric and its derivatives can be utilized as performance metrics of interest. The AoI quantitatively measures the relevance of information in the CC at a given moment in time. AoI’s upper bound for each update sent is measured by Peak Age of Information (PAoI). The mean PAoI across a sufficiently large amount of time and/or a large number of updates can be used as an optimization metric. The mean PAoI $M\left[A\right]$ for any system can be divided into two components: interarrival time $M\left[Y\right]$ and sojourn time $M\left[T\right]$ [5], that is,

(1) $$M\left[A\right]=M\left[Y\right]+M\left[T\right].$$

Recent reviews on AoI and PAoI analysis reported in [5,6] demonstrated that the analysis of these metrics in cellular systems is complicated (see Section 2). The principal difficulty stems not even from the lossy nature of wireless transmission medium [7,8], but mostly pertains to the scheduling process and orthogonal frequency-division multiple access (OFDMA) organization of the air interface. Indeed, the transmission time interval in both LTE and 5G New Radio (NR) is discrete and fixed, implying that models with batch service processes and batch arrivals must be utilized. The models are further complicated by the potential presence of autocorrelation in sensor measurements [9]. Theoretical foundations systems with autocorrelated batch arrival processes and batch service processes are rather limited [10,11].

In addition to the abovementioned challenges, the choice of service discipline is of special interest in the context of AoI/PAoI optimization. While last-come–first-served (LCFS) scheduling is theoretically optimal for minimizing AoI and PAoI metrics in single-source systems (see Section 2), its practical implementation in multi-source environments faces significant challenges. The co-existence of multiple sources requires additional constraints to ensure optimal AoI/PAoI performance across all sources. Furthermore, LCFS scheduling is rarely employed in contemporary networking architectures, with prioritized access mechanisms being more prevalent. To this end, there is the need to explore other service disciplines and cross-compare their performance.

The aim of this study is to analyze the PAoI in IIoT systems with multiple arrival processes of the same priority. To take into account the specifics of cellular systems with OFDMA technology support, this study proposes a model serving an arbitrary number of UEs, where each UE can be in one of two states: “regular” and “emergency”, where the “emergency” state corresponds to a higher intensity of update generation as compared to “regular”. The model is described as a queuing system (QS) in discrete time with a single server, limited buffer space, several batch input streams, and batch service process.

The main contributions of our study are as follows:

- We proposed a mixed analytical and simulation-based methodology for the analysis of the PAoI of a single traffic source in the presence of background traffic of the same priority in queuing systems with batch arrivals and batch services that are essential elements of modern cellular systems including 4G LTE and 5G NR.
- By utilizing the mean PAoI, we performed a cross-comparison of different scheduling strategies including last-come–first-served (LCFS), first-come–first-served (FCFS), random, and priority-based.
- We demonstrated that the performance of priority-based strategy is as good as the one of LCFS while when the numbers of sources grow, it actually outperforms the LCFS, and is thus recommended for use in modern cellular technologies serving state update IoT applications.

The remainder of this study is organized as follows. In Section 2, we do an overview of related work. In Section 3, we introduce the proposed system model. In Section 4, we formalize the queuing system. In Section 5, we describe the proposed solution approach. Numerical results are presented in Section 6. Finally, conclusions are presented in the last section.

## 2. Related Work

AoI and PAoI metrics were proposed in the beginning of 2010s. Specifically, in 2011, to quantify the freshness of available information about the state of remote systems, AoI and PAoI metrics were proposed [12]. These metrics allow a detailed characterization of the performance of mMTC and URLLC technologies and can be considered a quality of service (QoS) indicator.

Depending on their QoS requirements, mMTC and URLLC 5G services are characterized by different types of state update applications. AoI and PAoI metrics explicitly consider not only the network state but also the arrival time at the sender, characterizing the total time from the time an update is generated until it is received at the destination. This is a major factor distinguishing them from the latency.

Most of the initial studies were related to the applied areas of AoI and PAoI metrics usage. Specifically, researchers studied the impact of wireless channel characteristics and data transmission technology on AoI/PAoI performance. For example, ref. [13] considered intelligent transportation systems supported by unmanned aerial vehicles (UAVs) to deliver time-critical traffic updates to vehicles. The authors in [14] considered a new system model for estimating the average AoI for a URLLC wireless communication system having a Rayleigh block fading channel. In practical studies, AoI and PAoI metrics were considered for various scenarios that require timely updates. Note that most of these studies were performed using system-level simulation techniques.

AoI and PAoI metrics can be considered as a new class of performance metrics related to a queuing system. As a result, analytical studies started to appear [12,15,16,17,18]. At the initial phase, the simple queuing theory models were considered: M/M/1, M/D/1, and D/M/1 with the first-come–first-served (FCFS) service discipline [15]. Closed-form expressions for the average AoI and PAoI were obtained. The obtained insights allowed to specify the main areas of interest related to AoI/PAoI metrics including (i) developing special queuing disciplines and system configurations for AoI/PAoI minimization and (ii) relaxing the assumptions of simple queuing systems, leading to analytical models reflecting realistic arrival and service processes. Specifically, it was observed that, to minimize AoI and PAoI, last-come–first-served (LCFS) queuing discipline has to be utilized [16,18].

In [16], a system with several independent sources was considered, and time-averaged AoI characteristics were obtained. To allow for analytical tractability, the authors of [19] assumed that all sources generated packets at the same rate, which allows one to minimize AoI and PAoI. Systems with complex arrival and service processes are considered in [17,18,20,21,22]. In [18], special gamma-distributed service times were considered. In [17], the authors considered generally distributed uncorrelated service times, which is of interest for practical applications such as cellular systems and multi-homed wireless networks. In [20,21], discrete-time queuing systems were considered. In [20], the exact distributions of AoI and PAoI were numerically obtained using a matrix-geometric approach. In [22], the results were obtained for PAoI in a PH/PH/1 system, and the authors showed that bufferless and single-buffer queuing systems can effectively cope with the increased AoI that happens in FCFS queuing systems. It should be noted that the models for wireless systems has to account for retransmissions, resulting in complex packet service distribution. The abovementioned studies did not consider this feature, with the exception of the work [22]. For an exhaustive review of the literature on different models for AoI/PAoI analysis, we refer to [6].

As one may observe, most of the studies performed so far considered rather simple queuing systems that do not reflect specifics of arrival and service process in cellular systems. Specifically, only few studies considered batch arrivals and batch service that is natural for such systems due to frame-based scheduling process. On top of this, there is a lack of studies addressing systems with multiple sources. In our study, we relax these assumptions by considering a queuing system with batch arrivals and a batch service with multiple sources.

## 3. System Model

**Figure 1:**
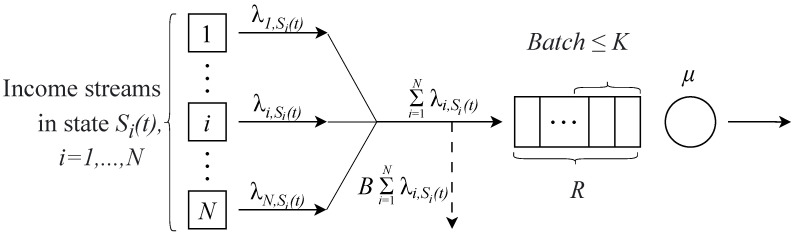
The considered queuing system model.

Let us consider the system model for the network supporting IIoT application as presented in Figure 1, similar to that considered in [4,23]. The model describes the uplink channel of the LTE/5G NR base station (BS) utilizing OFDMA multiple access scheme with a certain discrete transmission time interval (TTI). Network slicing functionality [24] is utilized to isolate state update traffic from the concurrent traffic.

An application of interest, consists of delivering state updates from remote UEs associated with certain equipment to the CC. As the TTI is finite, information from *N* UEs is sent in batches of updates and is collected in a buffer of BS. A maximum number of updates in the buffer is denoted as *R*.

Each UE can be in one of the two states j,j=1,2. For each UE, let the difference in states correspond to different update generation intensities denoted by $\lambda_{ij},\;i=\bar{1,N},\;j=1,2$, where *i* refers to the number of UEs, which generate updates and *j* refers to the state of the UE. We assume that, in state j=2, the UE has an update generation intensity that is higher than in state j=1. Furthermore, let $\lambda_{i}$ denote the mean update generation intensity for UE *i*, $\;i=\bar{1,N}$. $\lambda_{i}$ depends on the probabilities of switching between states for the *i*th UE.

Updates arriving at the system form an aggregated stream and are ordered based on the order they are generated at the UEs. Upon arrival, updates can be lost (dropped) if the buffer space is insufficient. The service time $\mu^{-1}$ for a batch of updates is fixed and equal to TTI, for example, 1 ms for 5G NR considered in this study. The service is also executed in batches of packets and the batch size in each TTI can be estimated by accounting for spectral efficiencies of UEs, as discussed in detail in [25]. In the following, we assume that its size is *K*. The service in each TTI consists of the following: (i) if there are no update packets in the buffer, nothing is transmitted to the service in this TTI; (ii) if the buffer has less than *K* updates, the batch of updates is formed from all the updates in the buffer; and (iii) if buffer has *K* or more updates, the batch of *K* updates is formed based on the utilized service discipline, and then the batch is served in a slot. To this end, we consider multiple service strategies including FCFS, LCFS, and others.

Finally, we assume that all packet transmissions are well protected by either forward error correction code (FEC code) or replication techniques.

## 4. Queuing Model Formalization

In this section, we formalize the queuing model and demonstrate that its analytical solution is only feasible for rather limited values of the number of UEs *N* and system capacity *K*.

### 4.1. Arrival Process

Because each of *N* UEs can be in either of the two states, let $S_{i}\left(t\right),\;i=\bar{1,N}$ denote the state of the *i*-th device immediately after the slot *t* starts. Furthermore, let $\alpha_{i}$ and $\beta_{i}$, $i=\bar{1,N}$ denote the probability of *i*-th UE switching from state 1 to state 2 and from state 2 to state 1, respectively. In this case, $\alpha_{i}$ and $\beta_{i}$ are given by

(2) $$\begin{array}{cc} \; & \alpha_{i}=P\left\{S_{i}\left(t\right)=2|S_{i}\left(t-1\right)=1\right\},\\ \; & \beta_{i}=P\left\{S_{i}\left(t\right)=1|S_{i}\left(t-1\right)=2\right\}. \end{array}$$

To account for all the UEs, let $S{\left(t\right)}^{⊤}=\left(S_{1}\left(t\right),\ldots ,S_{N}\left(t\right)\right)$ denote the vector of states of *N* devices at the start of slot *t*. For each UE stationary probability of being in state *j*, $P\left\{S(t)=j\right\},\;j=\bar{1,2}$ can be found as the stationary probabilities of the Markov chain.

The UEs generate updates at the end of each time slot. In each of the two states, the updates are generated in batches of size varying in accordance with Poisson distribution law with the mean given by $\lambda_{ij},\;i=\bar{1,N};j=1,2$, that is, $\lambda_{ij}$, $i=\bar{1,N};j=1,2$ denote the update generation intensity of *i*-th UE in state *j*, which corresponds to the mean size of the update batch sent to BS.

Consider the updates generated at an arbitrary slot, when at each UE, a batch of updates is generated. Let $W_{i}\left(t\right)$, $i=\bar{1,N}$ denote the size of the batch generated at *i*-th device at the end of time slot *t*. $W_{i}\left(t\right)$ is generated according to limited Poisson distribution, which is calculated as follows:

(3) $$P\left\{W_{i}\left(t\right)=k\right\}={\left(\sum_{l=0}^{R+K}\frac{{\lambda_{i,S_{i}\left(t\right)}}^{l}}{l!}\right)}^{-1}\frac{{\lambda_{i,S_{i}\left(t\right)}}^{k}}{k!},\;i=\bar{1,N},\;k=\bar{0,R+K}.$$

Generated updates from *N* UEs are ordered based on the order they were generated. The order is considered to be random; thus, each update has an equal chance of becoming the first or the last one in the batch. The batches generated at UE *i* are marked as

(4) $$\left\{\left(1,i\right)\ldots ,\left(k,i\right),\ldots ,(W_{i}\left(t\right),i)\right\},$$

where (k,i) is an update identifier, $i=\bar{1,N}$ is the UE to which an update belongs to, and $k=\bar{1,W_{i}\left(t\right)}$ is an arbitrarily assigned number of an update among those generated at UE *i*, $W_{i}\left(t\right)$ is the last update generated at UE *i* at slot *t*. The aggregated arrival batch generated from all the UEs is formed as

(5) $$\left\{\left(1,1\right),\left(1,2\right),\ldots ,\left(1,N\right),\ldots \left(W_{1}\left(t\right),1\right),\ldots ,\left(W_{i}\left(t\right),i\right),\ldots ,\left(W_{N}\left(t\right),N\right)\right\},$$

and the size of aggregated batch from all the UEs is $W\left(t\right)=\sum_{i=1}^{N}W_{i}\left(t\right)$.

Note that, when the number of UEs is rather high, the proposed approach may have high computational demands. To alleviate this challenge, one may represent the aggregated traffic from N-1 sources using a single arrival model and treat them as competing traffic for a tagged UE of interest. However, as the primary metric of interest is peak AoI, the targeted application is closer to an ultra-reliable low-latency communications (URLLC) service rather than a massive machine-type communications (mMTC) service in terms of AoI and latency requirements implying that the number of end sources is not expected to be large.

### 4.2. Queuing Dynamics

**Figure 2:**
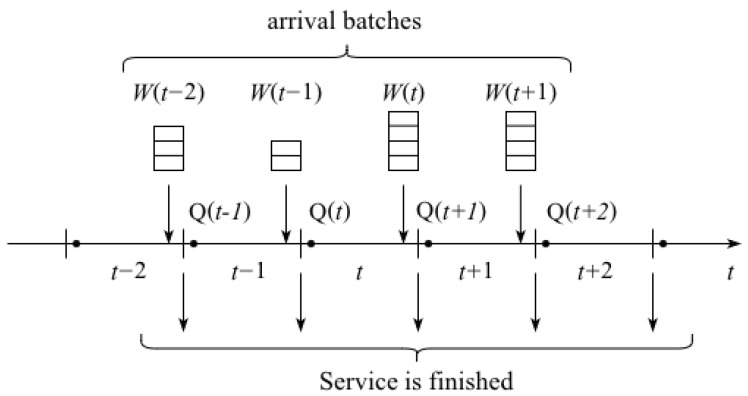
Time diagram of the considered system (D-BMAP^*X*^/D^*X*^/1/R).

The time diagram of the system is presented in Figure 2. Batches of generated updates arrive at the system at the end of the slot. Let Q(t) denote the occupied buffer space at the instance right after the slot *t* begins. Q(t) depends on the occupied buffer space at the previous slot, Q(t−1), the number of updates accepted in the previous slot, and the maximum size of service batch. Observe that, due to limited capacity, not all updates generated are accepted by the system. R−Q(t) denotes the buffer space accessible for updates at time slot *t*.

Let E(t) denote the number of updates accepted in slot *t*. Thus, for each slot *t*, E(t) is given by

(6) $$E\left(t\right)=\left\{\begin{array}{cc} 0, & Q(t)=R;\\ W(t), & W(t)\leq R-Q(t);\\ R-Q(t), & W(t)>R-Q(t). \end{array}\right.$$

When slot *t* starts, the buffer is checked for updates. If it is not empty, up to *K* updates form a batch and are sent for service. The number of updates forming a service batch depends only on the number of updates in the buffer. If the number of updates right after the previous arrival, Q(t−1)+E(t−1), is less than or equal to *K*, then all the updates in the buffer form the service batch, and thus, the occupied buffer space at the instance slot *t* starts, and Q(t) becomes zero. If, however, Q(t−1)+E(t−1), is greater than *K*, then only *K* updates form the service batch and the rest remain in the queue.

Thus, queuing dynamics Q(t) can be written as

(7) $$Q\left(t\right)=\left\{\begin{array}{cc} 0, & Q(t-1)+E(t-1)\leq K;\\ Q(t-1)+E(t-1)-K, & Q(t-1)+E(t-1)>K. \end{array}\right.$$

Substituting (6) to (7), we arrive at

(8) $$Q\left(t\right)=\left\{\begin{array}{cc} 0, & Q(t-1)+W(t-1)\leq K;\\ Q(t-1)+W(t-1)-K, & K<Q(t-1)+W(t-1)\leq R;\\ R-K, & Q(t-1)+W(t-1)>R. \end{array}\right.$$

### 4.3. Queuing Discipline

For the introduced QS, the following queuing disciplines are considered

- First-come–first-served (FCFS);
- Last-come–first-served (LCFS);
- Random choice (RAND);
- Priority service for updates from device *i* (PRIOR_*i*_).

Service discipline does not affect the size of a service batch. However, it affects the updates selected for transmission. The QS with FCFS as a service discipline will send to service up to *K* first updates in the buffer, regardless of their source UE. Similarly, with LCFS, the QS will schedule up to *K* last updates in the buffer. QS with service discipline RAND forms a service batch by choosing *K* random updates from the buffer. Finally, the PRIOR_*i*_ $i=\bar{1,N}$ discipline schedules up to $\left\lceil \frac{K}{2}\right\rceil$ updates from UE *i*, and the unoccupied space in the service batch is filled with updates from the buffer according to FCFS service discipline.

### 4.4. Analytical Approach

To analyze the introduced system analytically for the PAoI metric, one needs to account for all the *N* UEs, their respective state changes in between slots, and the number of updates in system. For N=1, this can be achieved as shown in [9], where a two-dimensional Markov chain simultaneously keeps track of the state of the arrival process and the number of updates in the buffer was utilized. Using this approach, the state-space of the system for arbitrary *N* is

(9) $$(S_{1}\left(t\right),\ldots ,S_{i}\left(t\right),\ldots ,S_{N}\left(t\right),Q_{1}\left(t\right),\ldots ,Q_{i}\left(t\right),\ldots ,Q_{N}\left(t\right)),$$

where $S_{i}\left(t\right),\;i=\bar{1,N}$, is the state of *i*-th UE immediately after slot *t* starts, $S_{i}\left(t\right)=\bar{1,2}$; $Q_{i}\left(t\right),\;i=\bar{1,N}$, is the buffer space occupied with updates from *i*-th UE immediately after the slot *t* starts, and $\sum_{i=1}^{N}Q_{i}\left(t\right)\leq R$. As can be observed, the dimensions of the state space are 2N, whereas the dimensions of the probability matrix are given by

(10) $$\left|Q\right|={\left(2^{N}\sum_{k=0}^{R}\frac{k!}{\prod_{i=1}^{N}k_{i}!}\right)}^{2},\;\sum_{i=1}^{N}k_{i}=k,$$

where the first factor accounts for all the combinations of the states of all the UEs in the system, and the second factor accounts for all the combinations of update packets in the buffer. Due to these constraints, we utilize the following simulation approach.

## 5. Simulation Model

In this section, we describe our simulation model. We begin with the simulation parameters, proceed with derivations of the metrics of interest, and finally discuss the simulation workflow.

### 5.1. Simulation Parameters

To simulate the considered system, several input parameters must be specified. The parameters used are listed in Table 1. Note that $\alpha_{i}$ and $\beta_{i}$, $i=\bar{1,N}$, are provided to the system as α and β, respectively. Similarly, $\lambda_{i,j}$, $i=\bar{1,N},\;j=\bar{1,2}$, are provided to the system as $\lambda_{j}$, respectively, $j=\bar{1,2}$.

For each generated update, several auxiliary parameters are stored in the simulation model. These parameters are further utilized to compute the statistical parameters of the metrics of interest. The auxiliary parameters are presented in Table 2.

Out of the parameters provided in Table 2, *n*, type, and $G_{n}$ are fixed at the instant when the update is generated at the UE. Then, the update is sent to the buffer, and if accepted, the $E_{n}$ value is set to True. If accepted, $F_{n}$ is modified at the time slot when the update is included in the service batch and sent for service. Once service is completed, information for previous updates is checked, and the $G_{n}^{prev}$ value is set based on the $G_{n}$ of the previously processed update of the same type value. If none of the processed updates have the same value of type, $G_{n}^{prev}$ is set to 0. For an update, PAoI, as calculated in [5], includes the interarrival and sojourn times.

From the simulation model data and the calculated auxiliary parameters, the final statistical parameters are collected and can be stored in any appropriate format. In addition to the information about each of the updates generated across the simulation model run, several additional metrics are calculated. Output parameters for each simulation are presented in Table 3.

### 5.2. Metrics of Interest

The number of generated, accepted, and lost updates is the direct result of the simulation model run. Update loss probability *B* is calculated as follows:

(11) $$B=\frac{L}{U}.$$

Update loss probability for the *i*-th type of updates, $B_{i},\;i=\bar{1,N}$ is calculated similarly to the update loss probability for the entire simulation run.

(12) $$B_{i}=\frac{L_{i}}{U_{i}},\;i=\bar{1,N}.$$

Mean interarrival time for a fixed type, $M_{i}\left[Y\right]$, is calculated as

(13) $$M_{i}\left[Y\right]=\sum_{n=1}^{U}Y_{n}\cdot I\left\{E_{n}\right\}\cdot I\left\{{type}_{n}=i\right\},\;i=\bar{1,N},$$

where I{condition} is an indicator function that is equal to 0 if the condition is not met, and is equal to 1 if the condition is met. Thus, $I\{E_{n}\}$ is a function testing if the update was accepted by the system, $I\{{type}_{n}=i\}$ is a function testing if the *n*-th update is of the *i*-th type. The indicator functions allow the system to filter all the generated updates, excluding those not accepted by the system and updates generated at other UEs.

Mean sojourn time for a fixed type, $M_{i}\left[T\right]$, is calculated similarly to the (13)

(14) $$M_{i}\left[T\right]=\sum_{n=1}^{U}T_{n}\cdot I\left\{E_{n}\right\}\cdot I\left\{{type}_{n}=i\right\},\;i=\bar{1,N}.$$

The mean PAoI for a fixed type, $M_{i}\left[A\right]$ is calculated as

(15) $$M_{i}\left[A\right]=\sum_{n=1}^{U}A_{n}\cdot I\left\{E_{n}\right\}\cdot I\left\{{type}_{n}=i\right\},\;i=\bar{1,N}.$$

Mean interarrival time for the whole system, $M\left[Y\right]$, in a simulation is given by

(16) $$M\left[Y\right]=\sum_{n=1}^{U}Y_{n}\cdot I\left\{E_{n}\right\}.$$

Finally, the mean sojourn time and mean PAoI across the whole system are calculated as follows:

(17) $$\begin{array}{c} M\left[T\right]=\sum_{n=1}^{U}T_{n}\cdot I\left\{E_{n}\right\},\;M\left[A\right]=\sum_{n=1}^{U}A_{n}\cdot I\left\{E_{n}\right\}. \end{array}$$

### 5.3. Simulation Workflow

**Figure 3:**
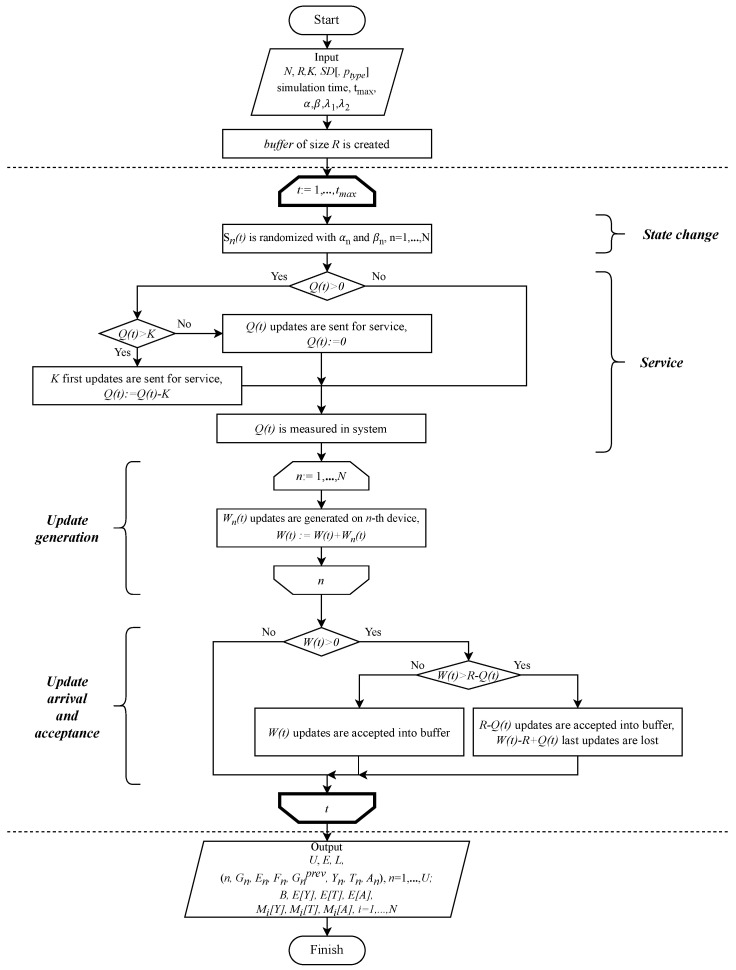
Flow diagram for the simulation model.

Simulation model for the considered system was written in Julia a general purpose programming language. Simulation model flow diagram is presented in Figure 3. First, the simulation model is parameterized with input parameters. Then, the main cycle begins. In between the dashed lines, the flow of the main cycle is described. Each iteration of the cycle is equal to one time slot (TTI) in the model. The cycle is repeated until the $t_{max}$ time slots are simulated. After the simulator run is finished, all the resulting parameters and statistics are returned and are stored for further usage.

## 6. Numerical Results

In this section, we present numerical results. We mainly concentrate on the impact of different service disciplines on the mean the PAoI metric. To gather the simulation data, a combination of sampling and replication techniques was employed [26]. For each set of parameters, the simulation was run 25 times, gathering 10^5^ observations in the steady state each time. The steady state period was detected using moving average statistics. The evaluated scenarios are listed in Table 4. Note that, for all scenarios, several parameters were fixed. The parameters used are listed in Table 5.

We will begin our numerical analysis, assessing how long it takes for the system to enter the steady state, allowing us to set the $t_{max}$ value for each simulation run. To this end, update generation rates for each arrival flow are set to match the condition $\sum_{i=1}^{N}\lambda_{i,1}=K$. As the update generation rate in the second state is greater than that in the first state, this condition effectively allows us to evaluate the mean PAoI under overloaded system conditions.

**Figure 4:**
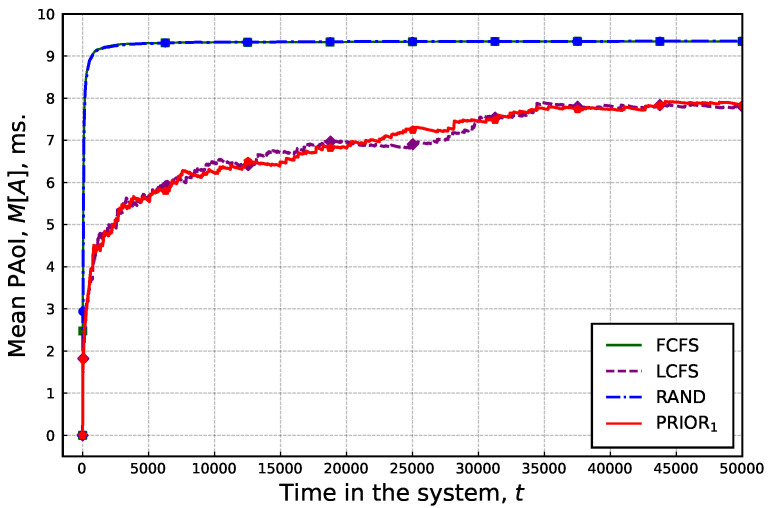
Mean PAoI as a function of time.

The obtained results are shown in Figure 4, where all the performed simulation runs are presented in the graph as a scatter plot, whereas the average values across all runs for each service discipline are plotted in colors. By analyzing the presented results, it can be observed that the service discipline affects not only the mean PAoI value, but also the way a mean PAoI behaves. Specifically, for RAND and FCFS service disciplines, the steady state is reached almost immediately after the simulation starting time at approximately 3000 time slots. For the other two considered disciplines, it takes approximately 10 times longer at 35,000 times slots to achieve the steady state, emphasizing the principal differences between the considered disciplines. In the following, we utilize the latter value as $t_{max}$.

**Figure 5:**
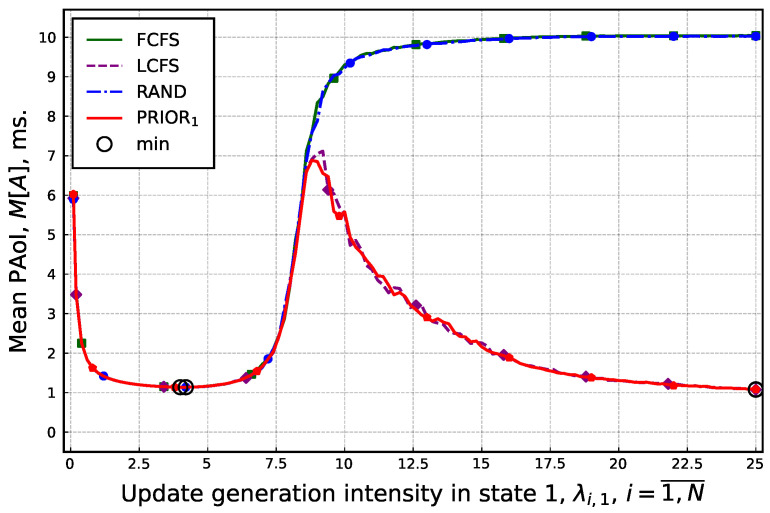
Mean PAoI as a function of the update generation rate.

Consider now the mean PAoI, M[A], as a function of the update generation rate in the first state of each arrival flow, $\lambda_{i1},\;i=\bar{1,N}$. To this end, for all the service disciplines, the average value across the runs is plotted in Figure 5. By analyzing the presented results, we observe that the mean PAoI demonstrates a different behavior for RAND and FCFS service disciplines as compared to the two other considered disciplines. However, the optimal update generation rate resulting in the minimal PAoI is independent of the service discipline and roughly corresponds to $\lambda_{i1}\approx 4$ updates/s. Thus, although the qualitative behavior of mean PAoI metric is different for different service disciplines, the optimal value of PAoI is insensitive to the choice of the service order.

**Figure 6:**
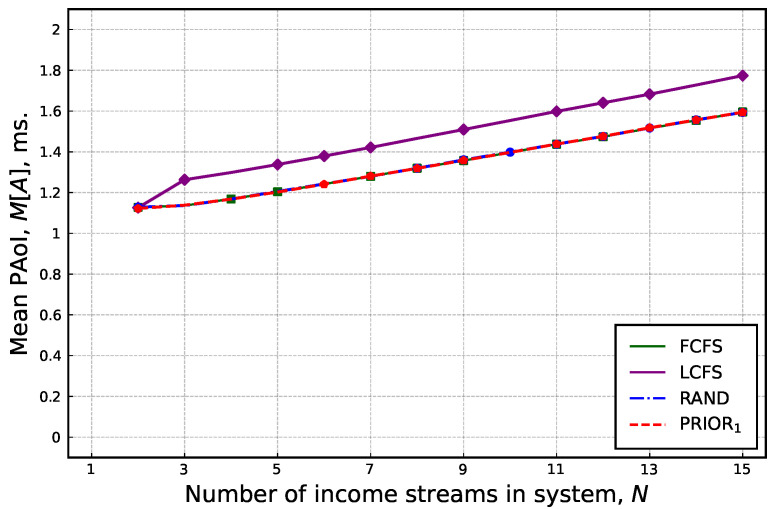
Mean PAoI as a function of number of UEs.

Having considered the aggregated performance, we now proceed with the performance of a single source. To this end, we first evaluate the mean PAoI as a function of the number of UEs in the system. In these experiments, we maintained a constant update generation rate. The obtained results averaged across all the runs are shown in Figure 6. The presented data show that the mean PAoI increases with the number of UEs. It can be observed that this does not occur because of the increase in the overall arrival rate of updates to the system, as it was kept constant. However, as the number of UEs increases, and thus, individual UE rates decrease, and the interarrival time between updates increases on average. We observed an increase in the mean PAoI values for all the service disciplines. As can be further observed, the mean PAoI values for PRIOR_1_ service discipline are greater than those for other disciplines. The rationale is that the priority allocated to an individual flow affects the performance of other flows.

**Figure 7:**
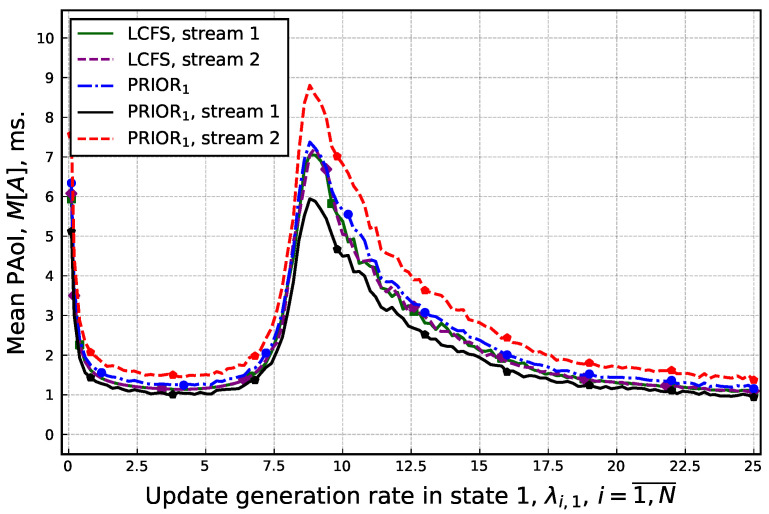
Mean PAoI for each of the UEs as a function of update generation rate.

Next, consider the impact of the update generation rate on individual source performance. Here, we analyze only LCFS and PRIOR_*i*_ SD as (i) among all the service disciplines, LCFS and PRIOR_*i*_ had a similar positive effect on mean PAoI across the whole system, and (ii) LCFS should have a consistent effect for individual UEs compared to PRIOR_*i*_, which should affect the individual UEs differently. The resulting data are shown in Figure 7. The presented data demonstrate that irrespective of the update generation rate and the utilized queuing discipline, the point minimizing the mean PAoI remains the same for both considered sources. Furthermore, we see that the low priority stream is characterized by a slightly higher mean PAoI as compared to the high priority one. However, this difference is rather negligible on the order of 10–15%. For the LCFS service discipline, the minimal mean PAoI expectedly remains similar.

**Figure 8:**
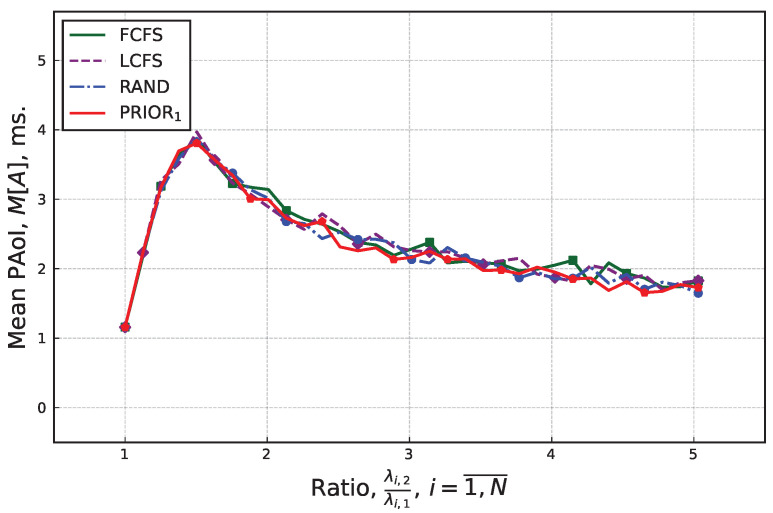
Mean PAoI as a function of ratio between update generation rates in different states.

Finally, we consider the mean PAoI for the case where we change the ratio between the update generation rates in the system. Practically, this implies that the variance of each source changes. The resulting data are shown in Figure 8. By analyzing the presented data, we see that, when the update generation rate in state 1 and $\lambda_{1}$ fixed, the variance of individual sources produces significant impact mean PAoI for all the considered service discipline. Specifically, the difference can reach three order of magnitude, as shown in Figure 8, where a significant spike is observed near the values of ratio ∼1.5. However, this impact is different across the considered values of the ratio between update generation rates in system.

In general, it is known that, for single-source systems, the use of an LCFS service strategy minimizes AoI and PAoI [27]. However, when multiple sources coexist in the system, additional restrictions are imposed to ensure that AoI/PAoI metrics are minimized [28]. On top of this, LCFS schedulers are not generally utilized in modern networking as opposed to prioritized access. In this context, the utilization of priorities provides ready-to-use alternatives for modern communications systems, as confirmed by our analysis. Specifically, we see that the performance of a priority-based strategy is as good as the one of LCFS while when the numbers of sources grow, it actually outperforms the LCFS.

## 7. Conclusions

In this paper, we proposed a model for mean PAoI assessment of correlated sources in cellular systems with batch arrivals and batch service. Motivated by the exponential growth in the state space of the modeling queuing system, we proposed to utilize a simulation based approach. We reported the model formalization as well as the algorithm utilized to model the system of interest.

Our analysis reveals that the PAoI performance of the priority-based strategy is as good as the one of LCFS while when the numbers of sources grow, it actually outperforms that of the LCFS. In contrast to the LCFS service strategies it also does not require additional mechanisms to ensure that the PAoI of a certain source of interest is optimized. Accounting for these facts and also recalling that priority-based service is generally supported by operational equipment, we may thus recommend it for use in modern cellular technologies serving state update IoT applications. However, we also note that allocating priority in service to one of the flows may affect performance of other flows even when the contribution of the priority flow is rather small.

We specifically note that the complexity of the considered model that includes batch arrivals and service processes, time-correlated multiple arrival processes and different service strategies required the use of mixed analytical and simulation methodology. One potential for the future work is to propose a fully analytical approach for priority-based service identified as a good alternative for systems with multiple arrival traffic sources.

## Figures and Tables

**Table 1:** Simulation input parameters.

Notation	Type of Data	Meaning
*N*	Integer	Number of update generating UEs in system
*R*	Integer	Buffer space in system
*K*	Integer	Maximum size of service batch
$\alpha_{i}$	Float	Probability of changing state from 1 to 2 for *i*-th UE, $i=\bar{1,N}$
α	Vector{Float}	Vector $\alpha ={\left(\alpha_{1},\ldots ,\alpha_{N}\right)}^{⊤}$
$\beta_{i}$	Float	Probability of changing state from 2 to 1 for *i*-th UE, $i=\bar{1,N}$
β	Vector{Float}	Vector $\beta ={\left(\beta_{1},\ldots ,\beta_{N}\right)}^{⊤}$
$\lambda_{i,j}$	Float	Mean number of updates in a slot from *i*-th UE in state *j*, $i=\bar{1,N},j=\bar{1,2}$
$\lambda_{j}$	Vector{Float}	Number of UEs in system, $\lambda_{j}=\left(\lambda_{1,j},\ldots ,\lambda_{N,j}\right)$
$t_{max}$	Integer	Simulation time measured in time slots

**Table 2:** Input parameters for each of the update simulated.

Notation	Type of Data	Meaning
*n*	Integer	Global sequential number of the update
$type_{n}$	Integer	Number of source UEs of *n*-th type, $type=\bar{1,N}$
$G_{n}$	Integer	Time slot when the update was generated, $G_{n}=\bar{1,t_{max}}$
$E_{n}$	Boolean	Indicator of the update acceptance to the system
$F_{n}$	Integer	Time slot when update was processed by the system
$G_{n}^{prev}$	Integer	Time slot when the previously accepted update of the same type was generated
$Y_{n}$	Integer	Interarrival time for the *n*-th update
$T_{n}$	Integer	Sojourn time for the *n*-th update
$A_{n}$	Integer	PAoI for the *n*-th update

**Table 3:** Output parameters for each of the updates simulated.

Notation	Type of Data	Meaning
$U_{i}$	Integer	Number of updates of the *i*-th type generated during a single run
*U*	Integer	Number of updates generated during the simulation model run
*E*	Integer	Number of updates accepted to the system
$L_{i}$	Integer	Number of lost updates of *i*-th type, $i=\bar{1,N}$
*L*	Integer	Number of lost updates
$B_{i}$	Float	Update loss probability for *i*-th type of updates
*B*	Float	Update loss probability
$M_{i}\left[Y\right]$	Float	Mean interarrival time for a fixed type, $i=\bar{1,N}$
$M\left[Y\right]$	Float	Mean interarrival time for the whole system
$M_{i}\left[T\right]$	Float	Mean sojourn time for a fixed type, $i=\bar{1,N}$
$M\left[T\right]$	Float	Mean sojourn time for the whole system
$M_{i}\left[A\right]$	Float	Mean PAoI for a fixed type, $i=\bar{1,N}$
$M\left[A\right]$	Float	Mean PAoI for the whole system

**Table 4:** Scenarios for system evaluation.

Scenario for Evaluation	*N*	$\lambda_{i1},\;i=\bar{1,N}$	$\lambda_{i2},\;i=\bar{1,N}$
Scenario №1. Mean PAoI as a function of time	3	10	$1.5\lambda_{i1}$
Scenario №2. Mean PAoI as a function of update generation rate	3	$\bar{0.1,15}$	$1.5\lambda_{i1}$
Scenario №3. Mean PAoI as a function of number of UEs	$\bar{2,15}$	12.6/N	$1.5\lambda_{i1}$
Scenario №4. Mean PAoI for each of the UEs as a function of update generation rate	2	6.15	$1.5\lambda_{i1}$
Scenario №5. Mean PAoI as a function of ratio between update generation rates in different states	3	12.6/N	$\bar{1,\;5}\cdot \lambda_{i1}$

**Table 5:** Fixed parameters for all the scenarios.

Parameter	Value
*R*	500
*K*	50
$\alpha_{i},\;i=\bar{1,N}$	0.225
$\beta_{i},\;i=\bar{1,N}$	0.675
Service discipline, SD	$\mathrm{FCFS},\;\mathrm{LCFS},\;\mathrm{RAND},\;{\mathrm{PRIOR}}_{1}$

## Data Availability

The original contributions presented in this study are included in the article. Further inquiries can be directed to the corresponding author.

## Abbreviations

The following abbreviations are used in this manuscript:

IoT	Internet of Things
IIoT	Industrial Internet of Things
AoI	Age of Information
PAoI	Peak Age of Information
QS	Queuing System
UE	User Equipment
CC	Control Center
OFDMA	Orthogonal Frequency-Division Multiple Access
NR	New Radio
BS	Base Station
TTI	Transmission Time Interval
FCFS	First-Come–First-Served
LCFS	Last-Come–First-Served
FEC	Forward Error Correction
RAND	Random choice
PRIOR	Priority Service
SD	Service Discipline
